# Interrelationships between Dietary Outcomes, Readmission Rates and Length of Stay in Hospitalised Oncology Patients: A Scoping Review

**DOI:** 10.3390/nu15020400

**Published:** 2023-01-12

**Authors:** Cecelia MacFarling Meure, Belinda Steer, Judi Porter

**Affiliations:** 1 Institute for Physical Activity and Nutrition, School of Exercise and Nutrition Sciences, Deakin University, Geelong, VIC 3220, Australia; 2 Nutrition and Speech Pathology Department, Peter MacCallum Cancer Centre, Melbourne, VIC 3000, Australia

**Keywords:** dietary intake, length of stay, hospital readmissions

## Abstract

Background: Poor food intake is an independent risk factor for malnutrition in oncology patients, and achieving adequate nutrition is essential for optimal clinical and health outcomes. This review investigated the interrelationships between dietary intakes, hospital readmissions and length of stay in hospitalised adult oncology patients. Methodology: Three databases, MEDLINE, Web of Science and PubMed were searched for relevant publications from January 2000 to the end of August 2022. Results: Eleven studies investigating the effects of dietary intakes on length of stay (LOS) and hospital readmissions in cohorts of hospitalised patients that included oncology patients were identified. Heterogenous study design, nutritional interventions and study populations limited comparisons; however, a meta-analysis of two randomised controlled trials comparing dietary interventions in mixed patient cohorts including oncology patients showed no effect on LOS: mean difference −0.08 (95% confidence interval −0.64–0.49) days (*p* = 0.79). Conclusions: Despite research showing the benefits of nutritional intake during hospitalisation, evidence is emerging that the relationship between intakes, LOS and hospital readmissions may be confounded by nutritional status and cancer diagnosis.

## 1. Introduction

Achieving adequate nutrition in a hospital setting is essential for optimal health outcomes in oncology patients. Unfortunately, oncology patients may fail to meet their nutritional requirements due to the side effects associated with disease and treatment. The consequences of failing to identify and manage inadequate dietary intakes include increased malnutrition risk and clinical complications (including pressure injuries, prolonged recovery, increased mortality and morbidity), as well as increased food waste and increased financial burden within the healthcare system [1].

Oncology patients frequently suffer from impairments to oral intakes which inhibit patient appetite and/or ability to eat and digest food leading to nutritional inadequacy. Tumour location, disease-related primary anorexia and cancer treatments (including chemotherapy, radiotherapy and surgery) [2,3,4,5] all contribute to these nutrition-related symptoms which may present as oral ulceration, xerostomia, poor dentition, intestinal obstruction, malabsorption, constipation, diarrhoea, nausea, vomiting, decreased intestinal motility, chemosensory alteration, weakness, fatigue, depression, uncontrolled pain and the side-effects from drugs [3,4,6,7,8,9]. Therefore, understanding the predictors of inadequate dietary intakes is an essential part of patient management and treatment. Several recent studies of hospitalised oncology patients have found that the predictors of decreased dietary intake include: being female, increased age, stage III or IV cancer, three-month unplanned weight loss and hospital stays longer than four days [5,9,10,11]. Socioeconomic issues may also impact food intake. Campos et al. [11] found that aside from clinical factors, work activity and economic class were associated with food intakes.

Hospitalisation may also contribute to inadequate dietary intake and decreased nutritional status. Hospital-related causes of inadequate dietary intake may be food service related, environmental, psychological and assistance related [12]. Issues surrounding the preparation, presentation and transport of food, as well as the accommodation of food preferences related to cultural or religious beliefs, may affect palatability and intake [13,14]. The timing of food service may not accommodate ward rounds or treatments, resulting in missed meals [15]. Psychological distress related to diagnosis and hospitalisation may also contribute to decreased intake [16]. Furthermore, an unidentified need for feeding assistance due to lack of mobility or cognitive issues may result in reduced intake [12].

Length of hospital stay (LOS) and hospital readmission rates are key indicators of the efficiency and effectiveness of hospital care and are significant factors for hospital management. The associations between dietary intakes (specifically, energy and protein) and these outcomes in hospitalised oncology patient populations are not well understood or quantified in the literature and may be influenced by standard treatment practices. The importance of dietary intake, its impact on malnutrition risk and subsequent clinical outcomes may also vary between cancer type and severity.

Longer LOS is associated with higher healthcare costs and is also subject to pragmatic, institutional or economic pressures [17,18]. A longer LOS may also pose an economic burden to the patient in the form of loss of employment and economic opportunity [19]. Hospital readmissions, particularly non-elective readmissions, are used as an indicator of morbidity, healthcare quality and efficiency [20,21,22]. Hospital readmissions are associated with increased healthcare costs, decreased patient quality of life and poorer clinical outcomes [20]. As a result, recent studies have focused on identifying predictors of non-elective hospital readmissions, largely to reduce healthcare costs.

Current European Society for Clinical Nutrition and Metabolism (ESPEN) guidelines for oncology care recommend robust nutrition care from the time of diagnosis running in parallel to antineoplastic treatments [23]. Achieving nutritional sufficiency reduces malnutrition risk and results in improved clinical outcomes, including shortened LOS and fewer non-elective hospital readmissions. However, structural and institutional barriers to meeting inpatient nutritional requirements and low adherence to ESPEN guidelines occur worldwide [23,24,25]. The purpose of this scoping review was to assess the evidence for interrelationships between dietary (energy and protein) intake and clinical outcomes, LOS and hospital readmissions, among adult hospitalised oncology patients.

## 2. Materials and Methods

### 2.1. Information Sources and Search Strategy

Three databases, MEDLINE, Web of Science and PubMed were searched for relevant publications from January 2000 to the end of August 2022. Database searches utilised the keywords: “dietary intake”, “energy intake”, “protein intake” and “nutritional intervention” together with “length of stay”, “length of hospital stay”, “LOS”, “duration of stay”, “unplanned hospital readmission”, “readmission rates” and “30-day readmission” with “cancer” or “oncology”. A literature search of Google Scholar using the same key words was also conducted. The reference lists of identified publications, including systematic reviews and meta-analyses were searched for additional publications. Included studies were restricted to peer-reviewed full text publications written in English.

### 2.2. Eligibility Criteria and Study Selection

The PICO (population, intervention, control and outcomes) framework was used to define eligibility criteria [26]. Journal articles were eligible if they were primary research studies that investigated oral dietary intakes (specifically protein and or energy intakes) among hospitalised adult cohorts that were comprised of exclusively oncological patients or included an oncological subgroup within the cohort, where LOS and/or hospital readmissions were primary or secondary outcomes. Papers were included if quantitative dietary intake data was collected and reported in kilocalories or kilojoules consumed for energy intakes and grams consumed for protein intakes. Studies where weighed or visual estimations of patient consumption were collected or where dietary data were collected using 24-h recall methods were included. Papers were excluded if dietary information was collected using subjective global assessment (SGA) or patient generated-subjective global assessment (PG-SGA) methods, as the food intake information is collected qualitatively through survey questions. The SGA typically asks the patient to rate their food intake during the last week or month as “unchanged”, “more than usual” or “less than usual” compared to their normal intake. Studies that investigated the effects of enteral or total parenteral nutrition were excluded.

Publication titles and abstracts were assessed against the selection criteria before the full texts of provisionally eligible studies were reviewed. Studies were excluded when there was not enough information on the dietary data or the study population composition, and where the clinical outcomes LOS and hospital readmissions were not studied.

### 2.3. Synthesis of Results

Nutrition intake data were converted to the same units: energy (kJ) and protein (g). A meta-analysis of randomised controlled trials reporting LOS was undertaken using Review Manager (RevMan) (computer program), version 5.4 (The Cochrane Collaboration, 2020) where mean and standard deviation were reported. The meta-analysis was undertaken using a random effects model [27], due to their being different protocols and participant groups. Statistical heterogeneity was assessed by the *I*^2^ value, where *I*^2^ values of 25%, 50% and 75% represent low, moderate and high heterogeneity [28]. Statistical significance was set at *p* < 0.05. Publication bias assessed by inspection of Funnel plots, and Eggers regression was not conducted as there were less than 10 studies.

A meta-analysis of randomised controlled trials reporting readmission data was not possible as this was only reported in the study by Schuetz et al. For other study designs, including cohort studies and retrospective analyses, as well as randomised controlled trials where mean and standard deviations were not reported, results of these studies were synthesised narratively.

## 3. Results

### 3.1. Study Selection

**Figure 1:**
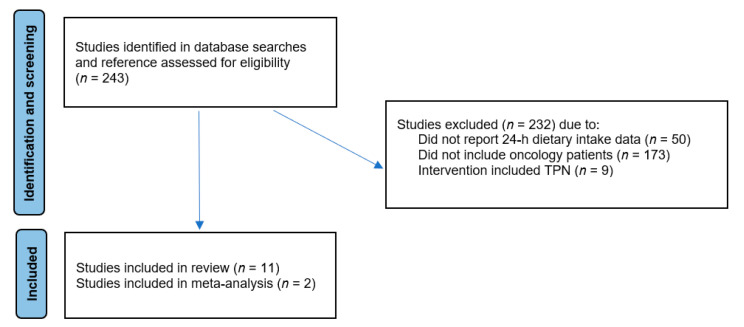
Flow diagram of study selection.

The database, reference list and grey literature searches identified 243 papers (excluding duplicates), shown in Figure 1. The titles and abstracts were screened against the eligibility criteria and 232 papers were excluded because they did not meet the eligibility criteria. Of these, 173 papers were excluded because they did not include information collected from oncology patients. A further fifty papers were excluded because they did not report daily dietary intake data reported in standard scientific units (for example, kcal/day, kJ/day for energy intakes and g/d or g/kg/day for protein), or because they did not collect dietary data using recalls, 5-point visual methods or weighed food methods. Nine papers were excluded because patient interventions included the use of total parenteral nutrition rather than oral supplementation or nutrition. A total of eleven peer review journal articles of discrete studies were eligible for inclusion in this review (Table 1).

### 3.2. Characteristics of Included Studies

The following data was collected for each study: first author, year of publication, study location/s, data collection period, study design, sample size, mean age of participants, sex (number/ratio), clinical diagnosis type of the cohort, nutritional assessment methods, energy intake data, protein intake data, LOS and hospital readmission rates.

The eleven studies included a total of 6634 patients (Table 1). Three studies were composed exclusively of oncology patients [32,36,39], while eight studies were composed of mixed-diagnosis hospital inpatients that included a subgroup of oncology patients [29,30,31,33,34,35,38]. The studies were highly heterogeneous, with differing study designs, intervention types and follow-up durations. Cohort size, mean age and sex, as well as primary and secondary outcomes also varied between studies. Additionally, the studies investigating mixed-diagnosis inpatient cohorts varied with respect to care settings, diagnoses, treatment types and relative percentages of patients in each sub-group. Among the oncology cohorts there were varied cancer diagnoses (general, haematological, and colorectal cancers).

### 3.3. Energy and Protein Intakes

Dietary energy and protein intakes were estimated using validated methods, including 24-h recalls, and 5-point visual scale methods, for all studies. Nutrition interventions assessing the dietary intakes in mixed-diagnosis hospitalised cohorts typically use a benchmark of patients achieving ≥ 75% recommended daily intakes (RDI) of protein and energy [29,31,33,35,38,39].

The nutrition interventions showed an increase in daily energy and protein consumption in all cases [30,31,33,34,35,36,37,38,39]. The interventions increased energy intakes by a minimum of 623 kJ per day (*p* = 0.006) [37] to 2000 kJ per day (*p* = 0.000) [33]. Interventions increased protein intakes, 9.6 g/day (*p* = 0.011) [31] to 23.7 g per day (*p* = 0.002) [37]. Sharma et al. [30] noted no difference between median daily protein intakes between intervention and control groups (IG 23.25 g vs. 24 g, *p* = 0.137) [30].

### 3.4. Length of Stay

**Figure 2:**
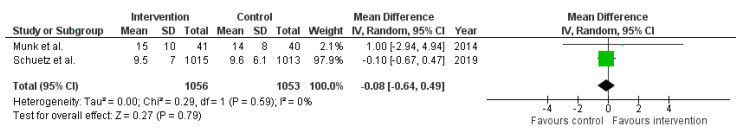
Meta-analysis of randomised controlled trials reporting length of stay [31,38].

The effect of dietary intake on length of hospital stay was investigated in ten studies. Meta-analysis (Figure 2) of the two studies reporting relevant length of stay data showed no effect between intervention and control groups: MD −0.08 (95% CI −0.64–0.49) days (*p* = 0.79). Low heterogeneity between these studies was evident (*I*^2^ = 0%).

No statistically significant relationship between dietary intakes and LOS was noted in six of the studies with mixed inpatient populations [29,31,32,33,37,38]. Conversely, four studies with a focus on oncology patients found an association between dietary intake and LOS [30,34,36,39]. Three of these studies [30,34,36] focused on specific oncology types (haematology or colorectal cancer) and treatment (surgical or chemotherapy) populations.

Sharma et al. [30] observed a shorter LOS associated with a nutrition intervention using a high-energy, high-protein oral supplement following colorectal surgery (median 6.5 days versus 9 days). However, the small cohort and LOS as a secondary outcome mean that the study was not powered to detect differences in LOS [30]. Furthermore, significant age differences between the intervention group (median 61.5 years) and control group (median 71 years) mean that age-related health effects on health and recovery may be a confounding factor. Similar findings were observed by Yeung et al. [36] using an enhanced recovery after surgery protocol (high protein) in colorectal cancer surgical patients (6.5 days versus 9.7 days). That study noted that patients who met >60% of their recommended protein goals in the first three days post-operative period were associated with a shorter LOS, despite oral intakes not differing between groups. However, the intervention group also had a significantly lower percentage of patients at risk of malnutrition (16% versus 33%) [36], which may independently impact LOS. Villar-Taibo et al. [34] noted a shorter LOS in haematological cancer patients with heightened malnutrition risks, where patients who met their recommended daily intakes had a shorter LOS of 3.5–4.5 days. However, the acute diagnoses of the cohort resulted in relatively long LOS (11.5 days) which may have allowed the intervention to have greater effects. Additionally, dietary recalls were self-administered and may have introduced memory bias. Hsieh et al. [39] found that LOS was shorter (7.9 days versus 14.7 days) in patients who had adequate intakes (≥80% RDI) versus patients who had inadequate intakes (≤50% RDI) among a general oncology population. However, a large number of the cohort also required nasogastric feeding.

In studies composed of general medical patients, Agarwal et al. [29] noted a longer median LOS (2 days) in patients who consumed ≤25% food intake compared to patients who consumed ≥50% offered foods; however, the overall percentage of food intake was not significantly associated with LOS [29]. That study observed a relationship between longer LOS and increased malnutrition risk, and noted that decreased food intakes during hospitalisation are an independent risk factor for malnutrition [29]. This observation was noted by several other trials in this review [37,38]. Ramos-Martinez et al. [37] noted that malnourished patients had a significantly longer median LOS (22.5 vs. 14 days). The relatively long LOS in that study may have allowed time to maximize effectiveness of the nutrition interventions, as the longer LOS may have allowed patients to adjust to hospital foods, increase intakes and improve clinical outcomes. Results associated with this longer LOS may not reflect current standards of care and may not be generalisable to all settings. Schuetz et al. [38] noted that >20% of patients were discharged prior to meeting their recommended nutrition goals, limiting the impact of the nutritional intervention.

### 3.5. Hospital Readmissions

Six studies assessed the relationship between dietary intake in hospitalised patients (including subgroups of oncology patients) and hospital readmissions, and found no statistically significant association [29,32,35,36,37,38]; however, only one of these studies [38] assessed non-elective readmission rates. Heterogeneity of study designs, for example, all-cause readmissions versus unplanned readmissions and length of time between discharge and readmission (30 days versus 90 days), as well as population differences, for example, mixed-diagnosis inpatients vs. oncology inpatients, may preclude generalisations.

Two studies of general hospital inpatient populations [29,32] observed a relationship between increased malnutrition or malnutrition risk and increased all-cause hospital readmissions. Agarwal et al. [29] noted that while there was no significant association between intakes and hospital readmissions, malnourished patients were found to have a higher rate of readmission (36%) compared to well-nourished patients (30%), suggesting that dietary intakes may play a role in reducing hospital readmission through reducing malnutrition risk [29]. Additionally, oncology patients have been found to have a higher rate of hospital readmission (43%) [29]. Calleja-Fernandez et al. [32] found that 47% of a haematology-oncology patient cohort were at risk of malnutrition, and furthermore, that increased malnutrition risk resulted in an increase of 30-day hospital readmissions from 8% to 31%.

## 4. Discussion

This review aimed to assess the evidence for interrelationships between dietary (energy and protein) intake and clinical outcomes, LOS and hospital readmissions, among adult hospitalised oncology patients. Key findings of this review are:

1. Studies found varied results for the relationships between dietary intakes and LOS. This is likely due to heterogeneity of the study designs (including randomised control trials, observational, or retrospective studies), interventions used, methodology for collecting dietary intake data (including 24-h recalls or staff-recorded 5-point visual records) and cohort demographics (including mixed hospital patients with oncology subgroups, where percentages of oncology patients varied from 6.6 to 100%). Five studies consisting of mixed diagnosis hospitalised patient cohorts, including two large studies, found no statistically significant relationship between improved dietary intakes and shorter LOS [29,31,32,33,38]. Conversely, five studies focusing predominantly on oncology patients found an association between increased dietary intake and shorter LOS [30,34,36,37,39].

2. No statistically significant relationship between increased dietary intakes and hospital readmission rates were noted in any of the studies where it was a measured outcome [29,32,35,36,37,38]. However, heterogeneity of study designs, for example, all-cause readmissions versus unplanned readmissions and length of time between discharge and readmission (30 days vs. 90 days), as well as population differences, for example, mixed-diagnosis inpatients vs. oncology inpatients, may preclude generalisations.

Few systematic reviews and meta-analyses focusing on the dietary intakes of oncology inpatients and LOS are available to compare with the conclusions of this review. Gupta et al. [19] noted that improved nutritional status was associated with shorter LOS in surgical patients with gastrointestinal cancers; however, this review focused on trials investigating nutritional support, not dietary intakes. Similarly, Deftereos et al. [40] noted decreased LOS was associated with increased pre-operative nutrition support. That review allowed for trials with a wider range of nutrition interventions, including enteral and parenteral nutrition, and found that the quality of evidence was very low [40]. Several recent systemic reviews and meta-analyses of general medical inpatients at malnutrition risk [41,42] have found no association between increased dietary intakes and LOS. However, Gomes et al. [42] did note that nutrition support was associated with greater benefits in the subgroup of patients with established malnutrition versus those that were identified as at nutritional risk. Gomes et al. [42] included a larger and more clinically diverse population, such as intensive care patients. Treatment of acute care patients is associated with longer LOS and very specific metabolic and nutritional needs not generalisable to oncology populations [29,38,42]. Feinberg et al. [41] included studies with more intensive nutritional interventions and therapies (enteral/parenteral nutrition) than included here.

This review suggests that an association between dietary intakes and LOS may be obscured when oncology patients are included with general medical patients due to the specific nutritional needs of oncology patients. We note that malnutrition risk varies with cancer diagnosis; consequently, the impact of nutritional interventions on clinical outcomes is likely to be diagnosis dependent, rather than generalisable across all oncology cohorts. Malnutrition and decreased nutritional status have been associated with longer LOS in general hospitalised patients [29,43], surgical patients [44,45] and oncology patients [19,46]. Poor dietary intakes during hospitalisation are a risk factor for malnutrition, and malnutrition is an independent risk factor for increased LOS [19,29,43].

In contrast to this review, Gomes et al. [42] noted an association between increased dietary intakes and a reduction in non-elective hospital readmissions (14.7% vs. 18.0%; risk ratio, 0.76; 95% CI, 0.60–0.96). Many studies were unable to include data where patients were admitted to other hospitals or other forms of inpatient care, including hospices. Patient presentations at emergency rooms were also not captured by the studies. Only one study included in this review differentiated between elective and non-elective readmissions; however, Schuetz et al. [38] found no association between non-elective readmissions and dietary intakes. Agarwal et al. [29] noted that oncology patients were readmitted at greater rates than the overall study population (43% vs. 30%, *p* = 0.032). Two studies [29,32] observed a relationship between increased malnutrition or malnutrition risk and increased hospital readmissions, despite not finding an association between readmissions and dietary intakes. Insufficient nutritional follow up after discharge has been identified as a cause behind the similar readmission rates between groups in nutrition intervention studies where inpatient dietary intakes were increased [35]. Finally, Munk et al. [35] posited that insufficient nutritional follow up post-discharge may explain similar readmission rates between intervention and control groups in some trials.

This review recognises that LOS and hospital readmissions are dependent on a multitude of factors that are unrelated to nutritional adequacy and dietary intakes. These may include financial, institutional and clinical pressures. Significant geographic variations in LOS occur globally, reflecting differences in the availability of hospital services [17]. Factors that influence access to hospital services may be attributed to variations in cultural norms around disease, death and hospitalisation, economic conditions that reduce the availability of hospitals, resources or advanced treatments, and distance (for example, rural vs. urban areas) which may lengthen LOS in some areas, while proving prohibitive to hospital admission in others [17]. A lack of available post-hospital care options, including hospice, rehabilitation, outpatient management programs or at-home nursing care, may increase LOS [17,47]. A temporal shift in LOS may occur for certain conditions, for example, as treatments for breast cancer have improved, a shorter LOS has become standard over time—in regions where advanced treatments are available [48]. Clinical predictors of longer LOS have been identified such as increased age, affected organs and two or more co-morbidities [17,49]. A study of Australian oncology patients found that psychological distress and being female were also associated with longer LOS [50].

Recent studies have shown that readmission rates among oncology patients vary between 13.8% to 30%, some of which are preventable [22,51,52,53]. Hospital readmission rates have been shown to vary by type of cancer (highest rates of non-elective readmissions occurred among bladder, pancreatic, ovarian and liver cancer patients), treatment (surgical vs. chemotherapy), disease stage, presence of co-morbidities, longer LOS, and increased age [20,54,55,56].

Clinical causes for non-elective readmissions among oncology patients include postoperative complications (for example, infection and surgical wound complications), poorly controlled pain, malnutrition or dehydration due to nausea, vomiting and/or diarrhoea, failure to thrive and lack of readiness for discharge [22,53,54]. An association was observed between a first chemotherapy cycle, inpatient intravenous fluid use and antiemetic prescription use and increased non-elective hospital readmissions in older oncology patients (>60 years). [51] Furthermore, an association between abnormal laboratory results (for example, haemoglobin, albumin, and sodium concentrations) and increased non-elective readmissions among older (>65 years) oncology patients has been found [57]. This suggests that early identification and treatment of clinical side effects plays an important role in decreasing non-elective readmissions.

A number of limitations to this review exist. Study selection, where papers were screened by one author, may have resulted in data being missed. Literature was limited to studies written in English and authors acknowledge additional foreign language material may present further evidence not included in this review. This review also included data from observational as well as controlled intervention trials, which may limit the strength of associations between dietary intakes and outcomes. Additionally, study design, including type of nutritional interventions, methods of analysis and choice of primary/secondary outcomes, varied between studies which limited comparisons of results. Finally, heterogeneity of study populations was considerable, and relative composition of study populations impacted results.

Research targeting the cost-effectiveness of providing timely nutritional support interventions for hospitalised oncology patients would provide valuable insights to clinicians. Additionally, the evaluation of other patient-centered outcomes, including quality of life, should also be considered in future research in this population.

The findings of this review indicate that the relationship between dietary intakes and clinical outcomes, LOS and hospital readmissions, is dependent on diagnosis and may be linked to nutritional status. However, both these outcomes are multifactorial and are also dependent on non-nutritional factors, including institutional ones, that could prove to be barriers to attaining nutritional adequacy during hospitalisation. Future research should consider nutrition interventions that optimise clinical outcomes through targeting oncology populations at greatest risk and prevalence of malnutrition.

## Figures and Tables

**Table 1:** Findings from included studies reporting relationships between dietary intakes and relevant clinical outcomes.

Author, Date, Location	Study Design	Population Details	Results
Agarwal et al., (2013) [29] Australia and New Zealand	Multicenter cohort (56 hospitals) Duration: *t* = 90 days Outcomes: in-hospital mortality, LOS and 30-day readmissions	Total *n* = 3017 Mean age = 65 ± 18 years 47% female Oncology *n* = 199 (6.6%)	Longer median LOS for malnourished patients = 15 d vs. 10 d Longer median LOS (2 d) with ≤25% intake LOS = 13 (2–158) d vs. LOS = 11 (2–119) d ≥50% intake (*p* < 0.0001). Food intake % was not significant Overall HR = 30%, malnourished patients 36% UHR vs. well-nourished patients 30% UHR (*p* = 0.001). No association between food intake and UHR Oncology HR:_total *n* = 35 (43%)
Sharma et al., (2013) [30] United Kingdom	Single-center, RCT Duration: *t* = 3.5 years Outcomes: HGS, LOS	Total *n* = 55 Median age: IG = 61.5 years, CG = 71 years 64% male Oncology: colorectal surgical	Average daily energy intake was higher in the IG: IG = 3421 kJ/d, CG = 2207 kJ/d No difference in average daily protein intakes: IG = 23.25 g/d, CG = 24 g/d No difference in post-operative HGS or bowel activity. Median LOS was shorter in the IG: IG = 6.5 d, CG = 9 d
Munk et al., (2014) [31] Denmark	Single-blinded RCT Duration: 18 weeks Secondary outcomes: body weight, HGS, LOS	Total *n* = 84 at nutritional risk IG: *n* = 41, CG: *n* = 40 Mean age = 74 years Oncology *n* = 29 Orthopedics *n* = 22 Urology *n* = 30	Mean energy intakes were 693 kJ higher in the IG (*p* = 0.8, not significant), mean energy intake per body weight was 20 kJ/kg significantly higher in IG (*p* = 0.013) Mean protein intakes were 9.6 d/d in IG (*p* = 0.011), mean protein intake per body weight was 0.2 g/kg significantly higher in IG (*p* = 0.003) No significant differences between LOS (mean ± SD) of (15 d ± 10 d) IG vs. (14 d ± 8 d) CG (*p* = 0.38).
Calleja-Fernandez et al., (2015) [32] Spain	Observational cohort study Duration *t* = 30 days Outcomes: LOS, HR, mortality	Total *n* = 73 at admission and *n* = 29 at day 7 (60.3% loss) Mixed cancer patients Observational, no intervention	No significant difference between energy or protein intakes between day 1 and day 7 (*p* = 0.12) Prevalence of malnutrition was 47.7% HR was greater for malnourished patients (35.1%) than well-nourished (8%) (*p* = 0.014)
Maunder et al., (2015) [33] Australia	Quasi-experimental pre-test, post-test cohort Paper menu (CG) vs. BMOS (IG) Duration: 2 × 48-h periods Secondary Outcomes: LOS	CG: *n* = 54 (75% response rate) IG: *n* = 65 (95% response rate) Oncology patients paper = 6, BMOS = 10 Mean age = 65.1 years 64% female	Mean energy intakes increased significantly between the CG = 6273 kJ/day and the IG = 8273 kJ/d (*p* = 0.000) Mean protein intakes increased significantly between the CG = 66 g/d and the IG = 83 g/d (*p* = 0.001) Mean LOS was −1 d IG vs. CG (*p* = 0.01) Mean LOS was 9.8 ± 9.7 d CG vs. 8.5 ± 11.9 d IG
Villar-Taibo et al. (2016) [34] Spain	Prospective 2-year study Intervention duration *t* = 1 week Outcomes: LOS and nutritional status	*n* = 792 patients screened 37.8% *n* = 218 at risk Haematology inpatients recruited (83% oncology) Median LOS: 11.5 d	After 7 days intake had increased from 80 to 90% of RDI (*p* < 0.001) Increase in energy of 407.4 kcal/d and 17.6 g/d protein Median LOS = 11.5 d A trend towards a shorter LOS (3.5–4.5 d fewer) was observed in patients with higher dietary intakes
Munk et al., (2017) [35] Denmark	RCT with comparison to a historical intervention group (HIG) Duration: Jan–Aug 2014 Secondary outcomes: HR	Total *n* = 91, HIC: *n* = 41, IG: *n* = 50 Mean age: 74 years 65% female Oncology patients: surgical and non-surgical	Mean energy intake was greater in the IG (6763 kJ/kg) vs. HIG (5814 kJ/kg) (*p* = 0.001) Mean protein intake was greater in the IG (63 g/kg) vs. HIG (53 g/kg) (*p* = <0.001) HR did not differ between groups: mean IG: 16 d; HIG: 14 d; no SD reported.
Yeung et al. (2017) [36] Canada	Prospective cohort study, 2-centre study Duration: March 2014–April 2015 Outcomes: LOS, HR	CG: *n* = 46, female 46%, mean age = 57 ± 13 years IG (ERAS): *n* = 69, female 39%, mean age = 61 ± 14 years Diagnoses: colon cancer, rectal cancer	Total mean protein intakes were higher in the IG (0.54 g/kg/d) compared to the CG (0.33 g/kg/d) (*p* < 0.02) Oral food intake did not differ between groups IG had shorter LOS 6.5 d vs. 9.7 d CG (*p* = 0.049) No differences in 30-day HR found (*p* = 0.31)
Ramos-Martinez et al. (2019) [37] Spain	Prospective one-year study (January–December 2016) Secondary Outcomes: LOS, HR	*n* = 133 well-nourished at admission *n* = 28 (21%) developed malnutrition during LOS total *n* = 276 Oncology *n* = 20 (71.8%) Mean age = 63.4 ± 18.5 years 60.7% male	Energy intakes increased by 623 kg/d after intervention Protein intake increased by 27.3 g/d Long median LOS = 22.5 d in malnourished patients (vs 14 d well-nourished) (*p* < 0.01) No difference in 30-d HR (*p* = 0.254)
Schuetz et al., (2019) [38] Switzerland	Pragmatic, investigator-initiated, open-label, non-blinded, non-commercial multicenter RCT April 2014-Feb 2018 Outcomes: UHR, LOS	Total *n* = 2088 CG: *n* = 1050 (-*n* = 35), mean age = 72.4 ± 14.1 years cancer *n* = 201 (20%) IG: *n* = 1038 (-*n* = 25), mean age = 72.8 ± 14.2 years Oncology: *n* = 173 (17%) Expected LOS > 4 days	Energy intakes were higher in the IG (6274 kJ/d) vs. CG (5061 kJ/d) Protein intakes were higher in the IG (57 g/d) vs. CG (47 g/d) No difference in mean ± SD LOS: IG: 9.5 ± 7.0 d vs. CG: 9.6 ± 6.1 d (*p* = 0.46) No difference in UHR between groups. IG: *n* = 89 (9%) vs. CG: *n* = 91 (9%) (*p* = 0.96)
Hsieh et al. (2020) [39] Taiwan	Retrospective study Outcomes: mortality and LOS	*n* = 111 (57% male) Mean age = 58 years Mixed cancer patients 3 groups based on energy intake IEI: <50% RDI, *n* = 28 MEI: 50–79% RDI, *n* = 47 AEI: >80% RDI, *n* = 36	Energy intakes increased (*p* < 0.001) AEI: increased from 6381 to 6588 kJ/kg/d, MEI: increased from 4910 to 5924 kJ/kg/d IEI: increased from 1158 to 4261 kJ/kg/d Protein intakes increased (*p* < 0.001) AEI: increased from 1.27 to 1.36 g/kg/d MEI: increased from 0.87 to 1.17 g/kg/d IEI: increased from 0.22 to 0.89 g/kg/d LOS was shorter in patients who had adequate intakes (7.9 d) vs. patients who had inadequate intakes (14.7 d)

AEI: Adequate energy intake, BMOS: bedside menu ordering system, CG: Control group, HGS: handgrip strength, HIG: historical intervention group, HR: hospital readmissions, IEI: inadequate energy intake, IG: Intervention group, LOS: length of stay, MEI: Moderate energy intake, RCT: randomised control trial, SD = standard deviation, UHR = unplanned hospital readmissions.

## Data Availability

Data available within referenced articles.
